# BDNF Alleviates Microglial Inhibition and Stereotypic Behaviors in a Mouse Model of Obsessive-Compulsive Disorder

**DOI:** 10.3389/fnmol.2022.926572

**Published:** 2022-07-12

**Authors:** Yuchong Luo, Xiao Chen, Chunren Wei, Hongyang Zhang, Lingyi Zhang, Lu Han, Ke Sun, Boxing Li, Shenglin Wen

**Affiliations:** ^1^Department of Psychology, The Fifth Affiliated Hospital, Sun Yat-sen University, Zhuhai, China; ^2^Guangdong Provincial Key Laboratory of Brain Function and Disease, Neuroscience Program, Zhongshan School of Medicine and the Fifth Affiliated Hospital, Sun Yat-sen University, Guangzhou, China; ^3^Department of Burn and Plastic Surgery, People's Liberation Army of China Rocket Force Characteristic Medical Center, Beijing, China

**Keywords:** obsessive-compulsive disorder (OCD), RU24969, microglia, brain derived neurotrophic factor (BDNF), trehalose

## Abstract

Obsessive-compulsive disorder (OCD) is a severe mental illness characterized by obsessions and compulsions. However, its underlying mechanisms remain to be elucidated. Recent studies have suggested that neuroimmune dysregulation is involved in the pathogenesis of OCD. To investigate the role of microglia in this disorder, we established a pharmacological mouse model by using the serotonin (5-HT) 1A/1B receptor agonist RU24969 to mimic monoamine dysregulation in OCD, and we examined the morphological and functional alterations of microglia in this model. We found that RU24969 treatment led to compulsive circling behavior in mice. Strikingly, we found that the density and mobility of microglia in the prelimbic cortex were much lower in RU24969-treated mice than in control mice. Moreover, the expression of cytokines and chemokines, including BDNF, IL-1β, IL-6, TNFα, CD80, CD86, MHC-I, and MHC-II, also decreased in RU24969-treated mice. Importantly, we found that injection of BDNF or induction of BDNF expression by trehalose completely reversed microglial dysfunction and reduced stereotypic behavior. These results indicate that microglial dysfunction is closely related to stereotypic behaviors in our mouse model of OCD and that BDNF could be an effective treatment for stereotypic behaviors.

## Introduction

Obsessive-compulsive disorder (OCD) is a severe mental illness whose main features include excessive invasive thoughts and irresistible impulsive behaviors that affect patients' lives (Abramowitz et al., 2009). The World Health Organization lists OCD as one of the 10 most handicapping conditions owing to its high incidence of 2–3% in the general population and its high rate of disability (Torres et al., 2006; Veale and Roberts, 2014). However, despite the high incidence of this disorder, its pathogenesis, treatment, and other characteristics remain poorly understood (Robbins et al., 2019). As with most mental disorders, the pathogenesis of OCD still puzzles the scientific community. Although scientists are progressively unraveling this mystery, treatment remains unsatisfactory owing to the many unexplained problems that remain. At present, the treatment of OCD consists of two main aspects: (1) drug therapy, with selective serotonin reuptake inhibitors (SSRIs) as the first-line drugs, and (2) psychotherapy, with cognitive behavioral therapy based on exposure and response prevention (ERP) as the first-line approach. When these treatments fail, surgery can be used, but it is often accompanied by severe side effects.

Therefore, it is necessary to explore the pathogenesis of OCD and develop new treatments. Recently, increasing evidence suggests that an abnormal neuroimmune function is critical for the pathogenesis of OCD (Swedo et al., 1998; Marazziti et al., 2018). Microglia, as the innate immune cells of the brain, coordinate effective inflammatory responses and play an essential role in maintaining the homeostasis of the central nervous system (Nayak et al., 2012; Ransohoff and Engelhardt, 2012). In a positron emission tomography (PET) study of OCD, the concentrations of the translocator protein (TSPO) in the prefrontal cortex, thalamus, striatum, and other regions were significantly increased in patients with OCD, indicating that microglia were activated in these patients (Attwells et al., 2017). Moreover, studies also showed that defective microglia were the basis of compulsive grooming in Hoxb8-mutant mice, and the excessive grooming behavior of these mice could be rescued by transplanting normal microglia (Greer and Capecchi, 2002; Chen et al., 2010; Trankner et al., 2019). All these pieces of evidence indicate that microglial abnormalities may be involved in OCD.

However, previous studies have focused only on correlating microglia-related molecules with compulsive behaviors. The exact morphological and functional changes in microglia and the feasibility of microglia-based treatment for OCD have yet to be established. In our study, given the critical role of the serotonin system in OCD, we used the serotonin (5-HT) 1A/1B receptor agonist RU24969 to induce OCD-like behaviors in mice. This model allowed us to examine the OCD-related morphological and functional changes in microglia using wild-type mice. To investigate the role of microglial dysfunction in stereotyped behaviors, we performed immunofluorescence, *in vivo* two-photon imaging, flow cytometry, and behavioral tests to evaluate the differences in microglial morphology and function between the control and RU24969-treated groups. Importantly, we also explored whether the elevation of BDNF levels could help reverse microglial dysfunction and alleviate stereotypic behaviors in this OCD mouse model.

## Materials and Methods

### Animals

All experiments were conducted in accordance with the guidelines for the Care and Use of Experimental Animals issued by the National Institutes of Health of the United States, and the experimental scheme was approved by the Animal Care and Use Committee of Sun Yat-sen University. In this experiment, two mouse strains, C57BJ6 and CX3CR1-GFP+/–, were used. To prevent the changing estrogen levels of female mice from influencing behavioral outcomes, only male mice (8–10 weeks old) were included in this study. All experimental mice were raised under standard laboratory conditions, including a controlled 12/12-h light–dark cycle, unrestricted access to food and water, a temperature of 22°C, a humidity level of 60%, and cages with corncob litter. Before the start of the experiments, the mice were maintained in the housing facility for at least a week to adapt to their environment; they were then randomized to the control and experimental groups.

To minimize the effects of environmental factors (such as sound, brightness, and human odor) on behavioral results, the experimenter handled the mice in a consistent manner every day for at least a week before starting the experiments; this preparatory handling ensured that the mice were familiar with the experimenter. The sample sizes were as follows: 8–10 mice per group for the behavioral experiments, 3–4 mice per group for the immunofluorescence experiment, three mice per group for the two-photon experiment, four mice per group for real-time quantitative PCR, 10 mice per group for initial flow cytometry, and four mice per group for flow cytometry after rescue.

### Chemicals

RU24969 (a selective agonist of the 5-HT 1A and 1B receptors, HY-16688; MedChemExpress, USA), BDNF (CM135, CHAMOT BIO, China), and trehalose (to promote the synthesis and release of BDNF in the brain (Perucho et al., 2016), PHR1344, SIGMA, USA) were dissolved in sterile saline before use. On the previous dose–response studies (Du et al., 2013; Siteneski et al., 2018; Chen et al., 2021), RU24969, BDNF, and trehalose were administered at 10 mg/kg (ip), 0.25 μg/mouse (icv), and 6 μg/mouse (icv), respectively.

### Behavioral Experiments

The mice were brought into the test room 1 h before the experiments to acclimate to the experimental environment. Before the experiments, the mice in the experimental group were injected with RU24969 (10 mg/kg), while the mice in the control group were injected with the same volume of saline. After 5 min of the injection, the two groups underwent the circling behavior test and open field test in the same environment.

### Circling Behavior Test

We built a device with an open field consisting of a non-porous opaque plastic box with a side length of 35 cm and a wall height of 25 cm. Circling behavior was assessed, as described in a previous study (Seibenhener and Wooten, 2015). The mice were placed in the center of the open field, and the duration and number of bouts of circling were measured over a 20-min period with TopScan version 3.0 (CleverSys, Inc., Reston, USA) and SuperMaze (XR-Xmaze, Softmaze, Shanghai, China). The circling calculation program in TopScan was used to quantify circling behavior. Within the set range of movement speed, when the mouse completed a circling angle of 360°, this movement was recorded as one circle. In mice, persistent circling is a stereotyped behavior, similar to compulsive behavior in OCD, which is also characterized by repeated, persistent behavior. We evaluated whether the mice had compulsive-like behavior by recording the number and total duration of circling bouts in the device.

### Open Field Test

An open field was used to evaluate anxiety behavior. Mice were placed in the center of the open field as described in the circling behavior test. The number of entries into the inner zone, the total time spent in the inner zone, and the total distance covered were determined over 10 min.

### Immunofluorescence

The mice were anesthetized with propofol (160 mg/kg, lipid emulsion, intraperitoneal (ip) injection) 2 h after acute injection to induce stable anesthesia without a righting reflex or corneal reflex (Cattano et al., 2008). The mice were then killed, perfused with 1 × phosphate-buffered saline (1 × PBS), and fixed with 4% paraformaldehyde (AR1068, Boster Biological Technology, Wuhan, China). The brain tissue was then removed, postfixed with 4% paraformaldehyde for 24 h, and dehydrated with 30% sucrose solution for 2 days. After dehydration, the brain tissue was embedded and frozen in optimal cutting temperature compound (OCT). The brain tissue was sectioned coronally into 40-μm sections using a freezing microtome (CM1950; Leica, Wetzlar, Germany). Before immunostaining, the OCT was washed from the brain slices with 1 × PBS. Next, the brain slices were permeabilized and blocked with blocking solution (1 × PBS containing 0.25% Triton X-100, and 5% donkey serum) for 1 h. The slices were incubated with anti-ionized calcium-binding adapter molecule 1 (Iba1) antibody (rabbit, 1:500, WAKO, catalog #019-19741) at 4°C overnight and washed three times with PBS. Afterward, the sections were incubated with Alexa Fluor 488 antibody (donkey anti-rabbit, 1:500, A21208; Invitrogen, Carlsbad, USA) at 37°C for 2 h and washed with PBS. The nuclei were counterstained with DAPI (Sigma) for 5 min, and the slices were then washed three times with 1 × PBS. The brain slices were imaged with a confocal laser scanning microscope (AxioScan.Z1) equipped with a 20 × (numerical aperture (NA) 1.25) objective; five slices through the bilateral prelimbic cortex (PrL) of each animal were randomly selected. The PrL regions were roughly outlined in accordance with a brain atlas, and their areas were measured. NIH ImageJ software was used to measure the fluorescence intensity of microglia, cell counts, and the average area of cell bodies in the PrL region. The cell density was calculated as the number of microglia divided by the total area of the outlined field (Favuzzi et al., 2021).

### Skull-Thinning Surgery

Skull-thinning surgery was performed, as described by Yang et al. (2010). The specific procedure is as follows. The mice were anesthetized with propofol (160 mg/kg ip) and placed on cotton pads; heating pads were used to maintain their body temperature. Erythromycin eye ointment was applied to the eyes of the mice to protect their vision by preventing dryness. Most of the hair on the scalp of each mouse was shaved off with a double-sided razor blade. Then, an incision was made along the midline of the scalp, extending approximately from the neck region (between the ears) to the frontal portion of the head (between the eyes). Subsequently, we carefully disrupted the fascia between the scalp and the underlying muscle and skull using a pair of spring scissors under a dissecting microscope. After the PrL region was located based on the brain atlas, we fixed a hollow-centered skull holder over the marked region, ensuring that the area to be imaged was exposed in the center of the opening. After the glue was completely dry, the skull of the target region was evenly thinned to ~20 μm using a skull microdrill with a bit diameter of 1~2 mm.

### Two-Photon Imaging *in vivo*

*in vivo* two-photon calcium imaging was performed following a previously described procedure (Yang et al., 2010; Sipe et al., 2016). We used the CX3CR1-GFP+/– strain of mice to perform the two-photon experiment. The microglia of the mice expressed green fluorescent protein, allowing us to observe microglial morphology directly under the microscope. Before the start of two-photon imaging, it was necessary to connect a dissecting microscope to a camera to record and mark the imaging area and locate the region based on the spatial orientations of blood vessels to quickly identify the same imaging area for repeat imaging. The mice were placed under a two-photon microscope and imaged with parameters of 254.6 × 254.6 μm, 1,024 × 1,024 pixels, and a 0.69 μm step to form a z-stack image. The timeline for imaging was as follows: images were captured at two time points 15 min apart (t_1_, t_2_) before administration and another two time points 15 min apart (t_3_ and t_4_) beginning 5 min after administration. After completing the imaging, we gently removed the skull holder and the residual glue from the skull, disinfected the skull and scalp with iodophor, and then sutured the scalp with 6-0 silk. The mice were placed in separate cages until fully awake, after which they were returned to their original cages.

### Microcannula Implantation for Intraventricular Injection

Under deep anesthesia, the mice were fixed in a stereotaxic apparatus (RWD), the hair was removed from an area spanning from the ears to the eyes, and the exposed craniofacial skin was disinfected with iodophor. Eye ointment was applied to the eyes, as described before. A longitudinal opening was cut along the midline of the scalp with ophthalmic scissors to provide unobstructed access to the skull. A microcatheter (OD = 0.48 mm, C = 2.5 mm, RWD) was stereotaxically positioned in the lateral ventricle at the following coordinates relative to the bregma: AP, −0.3 mm; ML: 1.0 mm; DV, −2.5 mm. The microcatheter was fixed to the skull with dental cement and steel screws (M1.0 × L2.0 mm, RWD). The catheter cap (OD = 0.2 mm, G = 0.0 mm, RWD) was screwed into the microcatheter. The mice were housed individually in cages and allowed to recover for 7 days before infusion.

### Intraventricular Injection

The injection system consists of an injection tube (OD = 0.3 mm, C = 2.5 mm, G = 0.0 mm, RWD, filled with mineral oil) to be inserted into the cannula. The infusion procedure was performed 90 min before the start of the circling behavior experiment. The mice remained conscious and could move freely during the infusion. The cap was removed from the microcatheter for the infusion. The internal injection tube was inserted into the microcatheter and fixed in place with a screw (OD = 5.5 mm). The infusion volume was 3 μL. After each infusion, the tube was kept in the microcatheter for 5 min. When the procedure was complete, the animal was returned to its cage and provided with food until the experiment began.

### Preparation of a Single-Cell Suspension

Microglial cells were isolated using a previously described procedure (Hammond et al., 2019). In brief, the mice were killed and transcardially perfused with ice-cold Hanks' balanced salt solution (HBSS). The fresh brain tissues were quickly removed from the mice and placed on ice. The brain tissues were minced using a razor blade and then homogenized in ice-cold HBSS with a Dounce homogenizer, using the loose and tight pestles 20 times each while simultaneously rotating the pestles. The cell suspension was filtered through a 70-μm cell strainer to obtain a single-cell suspension and centrifuged at 500 × *g* for 8 min. The cell pellets were resuspended in ice-cold 40% Percoll diluted in HBSS and then spun for 30 min at 2,000 rpm with full acceleration and braking. The microglia accumulated in pellets at the bottoms of the tubes, and the Percoll and myelin were removed by vacuum suction. Each cell pellet was washed with 5 mL of ice-cold HBSS and centrifuged for 8 min at 500 × *g* at 4°C for flow cytometry.

### Flow Cytometry

Flow cytometry was used to evaluate the expression of CD80, CD86, MHC-I, and MHC-II in microglia. Single-cell suspensions were stained for 30 min on ice with ice-cold fluorescence-activated cell sorting (FACS) buffer (0.5% BSA, 1 mM EDTA, in 1 × PBS) containing anti-CD11b (Pecy7), anti-CD45 (AF700), anti-MHC-I (Percp5.5), anti-MHC-II (AF647), anti-CD80 (Pacific blue 450), anti-CD86 (BV605), live/dead (BV510), or matched isotype control antibodies from BioLegend at a 1:150 dilution. Flow cytometry analysis was performed using an LSRFortessa analyzer (BD) and FlowJo software (version 7.6.5; Treestar, Ashland, OR).

### Real-Time Quantitative PCR

Microglia were isolated from mouse brain tissue by magnetic-activated cell sorting (MACS) using magnetic-bead-conjugated anti-CD11b antibodies (Miltenyi Biotec, Auburn, CA) (Kumar et al., 2016). Total RNA was then extracted from the selected microglia using an RNeasy Plus Mini Kit (Qiagen, USA) following the manufacturer's instructions. RNase-free DNase sets (Qiagen) were used for DNase digestion during RNA purification. The quality and quantity of RNA isolated from tissues were evaluated by using a Nanodrop spectrophotometer, and reverse transcription PCR was performed using PrimeScript RT kits (Takara, Dalian, Liaoning, China). Real-time quantitative PCR (qPCR) was performed on an Opticon 2 thermal cycler (Bio-Rad, USA) with a FastStart Universal SYBR Master Mix for the indicated genes (Roche, USA). PCR primers were designed by Primer 5.0; the sequences are listed in the following text. Each sample was analyzed in duplicate with GAPDH as an internal control. Relative mRNA expression was calculated relative to the respective GAPDH Ct values and expressed as 2^−ΔΔ^*Ct*.

BDNF F: 5′-ACGTATTAGCGAGTGGG-3′,

R: 5′-ATGGGTAGTTCGGCATT-3′;

TNFα F: 5′-CGTCAGCCGATTTGCTATCT-3′,

R: 5′-CGGACTCCGCAAAGTCTAAG-3′;

IL-6 F: 5′-ATGGATGCTACCAAACTGGAT-3′,

R: 5′- TGAAGGACTCTGGCTTTGTCT-3′;

IL-1β F: 5′- GCCCATCCTCTGTGACTCAT-3′,

R: 5′- AGGCCACAGGTATTTTGTCG-3′;

GAPDH F: 5′- CAAAATGGTGAAGGTTCGGTGTG-3′,

R: 5′-TGATGTTAGTGGGGTCTCGCTC-3′.

### Statistical Analysis

GraphPad Prism (version 8.0) was used for statistical analysis. Where appropriate, data were tested for significant differences using Student's *t*-test (unpaired *t-*test or paired *t-*test with Welch correction) or ANOVA. Data are reported as mean ± SEM. *P* ≤ 0.05 was considered statistically significant.

## Results

### RU24969 Induced Stereotyped Behavior in Mice

**Figure 1:**
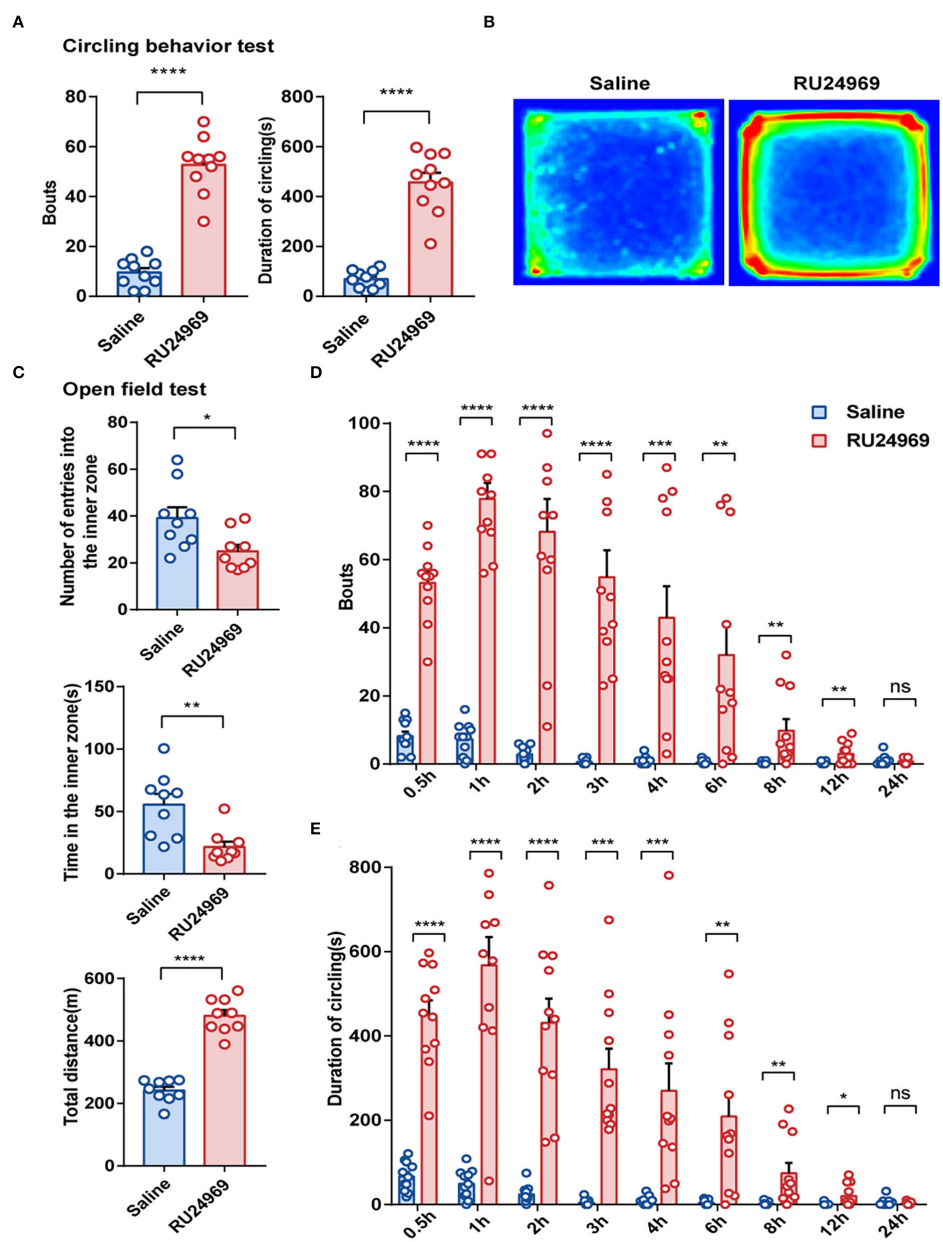
RU24969 induced mice to exhibit acute stereotyped behavior, and the effect disappeared within 24 h. **(A)** Intraperitoneal injection of RU24969 in mice significantly increased the duration and number of bouts of circling in the device (*n* = 10 mice; bouts: 9.5 ± 1.74 vs. 52.7 ± 3.55, *t*_18_ = 10.93, *P* < 0.0001; duration: 68.8 ± 11.41 vs. 457.1 ± 37.98, *t*_18_ = 9.793, *P* < 0.0001). **(B)** Heatmap of the mouse circling behavior experiment. **(C)** In the open field test, experimental mice showed anxious behavior and a state of hyperlocomotion (*n* = 9 mice; number: 39.11 ± 4.656 vs. 25 ± 2.749, *t*_16_ = 2.61, *P* < 0.05; time: 55.56 ± 8.531 vs. 21.67 ± 4.273, *t*_16_ = 3.552, *P* < 0.01; distance: 241.4 ± 12.14 vs. 480.8 ± 18.45, *t*_16_ = 10.84, *P* < 0.0001). **(D,E)** Statistical analysis of the number bouts and the total duration of circling behavior in mice 24 h after ip administration (*n* = 11 mice; bouts: 0.5 h: 8.182 ± 1.347 vs. 53.18 ± 3.247, *t*_20_ = 12.8, *P* < 0.0001; 1 h: 7.273 ± 1.471 vs. 77.82 ± 4.723, *t*_20_ = 14.26, *P* < 0.0001; 2 h: 2.818 ± 0.6581 vs. 68.18 ± 9.645, *t*_20_ = 6.71, *P* < 0.0001; 3 h: 0.6364 ± 0.2787 vs. 54.82 ± 7.884, *t*_20_ = 6.868, *P* < 0.0001; 4 h: 1 ± 0.3568 vs. 42.91 ± 9.279, *t*_20_ = 4.513, *P* < 0.001; 6 h: 0.4545 ± 0.2073 vs. 32 ± 9.184, *t*_20_ = 3.434, *P* < 0.01; 8 h: 0.2727 ± 0.1408 vs. 9.818 ± 3.33, *t*_20_ = 2.864, *P* < 0.01; 12 h: 0.1818 ± 0.122 vs. 3 ± 0.9723, *t*_20_ = 2.876, *P* < 0.01; 24 h: 1 ± 0.4472 vs. 0.7273 ± 0.2371, *t*_20_ = 0.5388, *P* = 0.5960; duration: 0.5 h: 66.93 ± 10.49 s vs. 449.1 ± 35.28 s, *t*_20_ = 10.38, *P* < 0.0001; 1 h: 49.84 ± 10.18 s vs. 567.6 ± 67.26 s, *t*_20_ = 7.611, *P* < 0.0001; 2 h: 24.08 ± 6.361 s vs. 431.7 ± 56.96 s, *t*_20_ = 7.112, *P* < 0.0001; 3 h: 4.611 ±2.298 s vs. 321.3 ± 49.04 s, *t*_20_ = 6.45, *P* < 0.0001; 4 h: 8.534 ± 3.168 s vs. 270.2 ± 65.22 s, *t*_20_ = 4.007, *P* < 0.001; 6 h: 4.062 ± 1.808 s vs. 208.8 ± 54.74 s, *t*_20_ = 3.739, *P* < 0.01; 8 h: 2.134 ± 1.276 s vs. 74.44 ± 24.61 s, *t*_20_ = 2.935, *P* < 0.01; 12 h: 1.018 ± 0.9163 s vs. 20.51 ± 8.146 s, *t*_20_ = 2.378, *P* < 0.05; 24 h: 5.129 ± 2.905 vs. 2.708 ± 1.052, *t*_20_ = 0.7836, *P* = 0.4424). Data are presented as the mean ± SEM. Data were analyzed using a two-tailed Student's *t*-test. ^*^P < 0.05, ^**^P < 0.01, ^***^P < 0.001, ^****^P < 0.0001, ns stands for “not significant”.

previous study showed that RU24969 could induce stereotyped behavior, especially repeated circling behavior (Chen et al., 2021). In this study, we obtained consistent results. In the circling behavior test, the RU24969 group circled more times and for a longer total duration than the saline group; the bouts of circling and the duration of circling of RU24969 mice were significantly increased after the mice were treated with RU24969 (Figures 1A,B). In addition, the mice treated with RU24969 showed anxious behavior and high motor activity. In the open field test, although the number of entries into the inner zone and the time spent in the inner zone of the open field were significantly lower in the RU24969 group than in the control group, the total distance of exercise was significantly higher in the RU24969 group than in the control group (Figure 1C).

To further explore the duration of stereotyped behavior induced by RU24969, we conducted circling behavior tests at several time points within 24 h after ip injection of saline or RU24969. The results showed that 12 h after injection, the number of circling bouts and the duration of circling were significantly higher in the RU24969 group than in the saline group, but there was no significant difference in the number or total duration of circling bouts between the two groups after 24 h (Figures 1D,E). These results indicate that RU24969 could consistently induce OCD-like behaviors. The duration of RU24969-induced OCD-like behavior in mice was at least 12 h, and the drug effect gradually subsided as the blood drug concentration decreased.

### Density and Motility of the Microglia in the PrL Were Reduced After RU24969 Treatment

**Figure 2:**
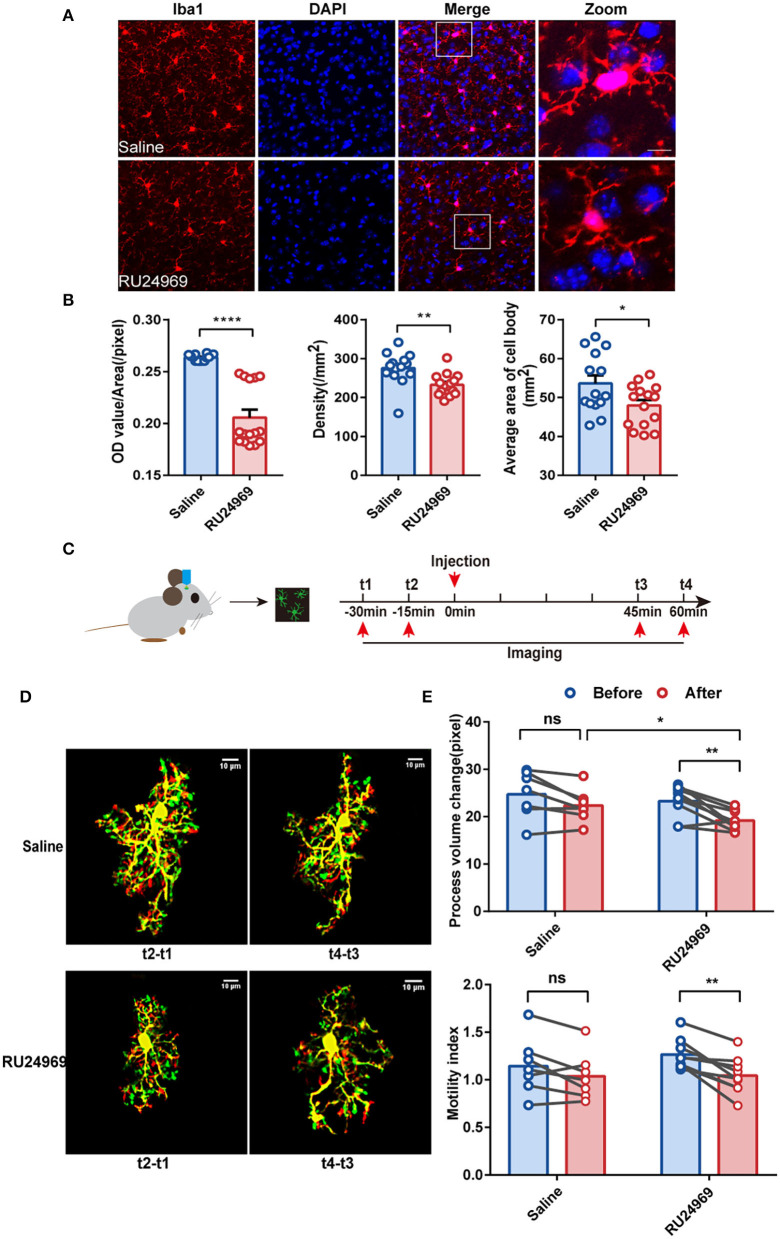
RU24969 resulted in decreased activity and motility of microglia. **(A)** Representative images of Iba1+ microglia in the PrL of mice in the saline group and RU24969 group. Scale bar: 10 μm. **(B)** Quantification of the fluorescence intensity, density, and average area of the cell bodies of Iba1+ cells in the two groups (*n* = 3 or 4 mice; 3–5 brain slices were selected for each mouse. Fluorescence intensity: 0.2636 ± 0.0007976 vs. 0.2058 ± 0.007571, *t*_27_ = 7.331, *P* < 0.0001; density: 275.5 ± 11.22 vs. 232.7 ± 7.257, *t*_27_ = 3.241, *P* < 0.01; average area of the cell body: 53.63 ± 2.036 vs. 47.94 ± 1.389, *t*_27_ = 2.336, *P* < 0.05). **(C)** Schematic diagram of the two-photon experimental design. **(D)** Representative images of microglia in the PrL brain region of mice in the two groups. Scale bar: 10 μm. **(E)** Quantification of the microglial motility index and process volume (paired *t* test, *n* = 3 mice, 2–3 cells per mouse, 7 cells in the control group, and 9 cells in the RU24969 group. Saline group: process volume change: *P* = 0.0530 > 0.05; motility index: *P* = 0.0959 > 0.05. RU24969 group: process volume change: *P* = 0.0034 < 0.01; motility index: *P* = 0.0012 < 0.01). Data are presented as mean ± SEM. Data were analyzed using a two-tailed Student's *t*-test. ^*^P < 0.05, ^**^P < 0.01, ^****^P < 0.0001, ns stands for “not significant”.

An increasing number of studies have shown that the prefrontal cortex (PFC) plays a crucial role in the pathogenesis of OCD (Ahmari and Rauch, 2022). As a part of the PFC, the PrL is located in a shallow cortical position and is closely related to the pathogenesis of psychiatric disorders (Seo et al., 2017; Joffe et al., 2019). To investigate the morphological changes in microglia after induction of OCD-like behavior, we first used immunofluorescence to compare the density of microglia in the PrL (Figures 2A,B) between the saline group and RU24969 group. Iba1 is a calcium-binding protein expressed specifically by microglia in the central nervous system. Our results showed that the fluorescence intensity [optical density (OD) value/area] of Iba1 in the PrL was significantly lower in the RU24969 group than in the control group. In addition, the density of microglia and the average area of the cell body decreased significantly in the PrL of the RU24969 group (Figures 2A,B).

To investigate the morphological changes in microglia in real time, we utilized a two-photon microscope to examine the motility of the microglial processes in the two groups (Figure 2C). The results showed that the changes in the process volume and the motility index were significantly reduced in the RU24969 group. However, there was no significant change in the saline group (Figures 2D,E). Together, these results indicate that the overall process volume and motility of microglia were reduced in the OCD model.

### Expression of Microglial Cytokines and Chemokines Was Decreased After RU24969 Treatment

**Figure 3:**
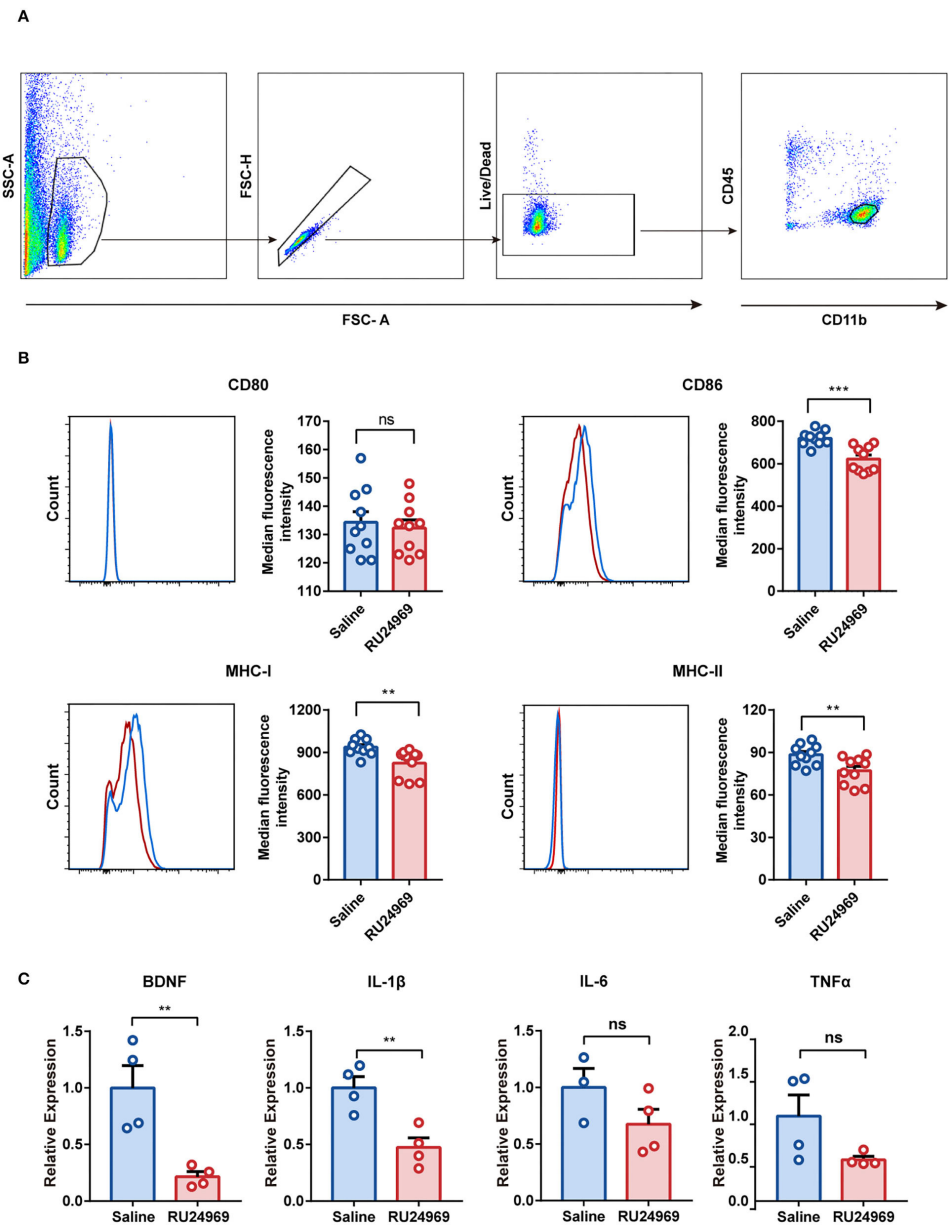
RU24969 resulted in decreased microglial function. **(A)** Microglia were isolated by flow cytometry according to the difference in surface antigen markers. **(B)** Quantitative comparison of the median fluorescence intensity of CD80, CD86, MHC-I, and MHC-II in microglia between the two groups (*n* = 10 mice, CD80: 134.3 ± 3.751 vs. 132.3 ± 2.868, *t*_18_ = 0.4236, *P* = 0.6769; CD86: 720.1 ± 10.88 vs. 622.3 ± 18.96, *t*_18_ = 4.475, *P* < 0.001; MHC-I: 936.9 ± 18.14 vs. 824.2 ± 30.74, *t*_18_ = 3.158, *P* < 0.01; MHC-II: 88.4 ± 2.288 vs. 77.08 ± 3.072, *t*_18_ = 2.955, *P* < 0.01). **(C)** Expression of BDNF, IL-1β, IL-6, and TNFα in microglia (*n* = 4 mice; one outlier was discarded from the IL-6 dataset. BDNF: 1 ± 0.1955 vs. 0.2158 ± 0.04455, *t*_6_ = 3.911, *P* < 0.01; IL-1β: 1 ± 0.09866 vs. 0.4733 ± 0.08598, *t*_6_ = 4.025, *P* < 0.01; IL-6: 1 ± 0.1684 vs. 0.6752 ± 0.1326, *t*_5_ = 1.54, *P* = 0.1842; TNFα: 1 ± 0.2486 vs. 0.4874 ± 0.03895, *t*_6_ = 2.037, *P* = 0.0878). Data are presented as mean ± SEM. Data were analyzed using a two-tailed Student's *t*-test. **P < 0.01, ***P < 0.001, ns stands for “not significant”.

To investigate the functional changes in microglia in an OCD mouse model, we analyzed the expression of microglial surface molecules, including CD80, CD86, MHC-I, and MHC-II, by flow cytometry (Figure 3A). Compared with the control group, the RU24969 group showed significantly reduced expression of CD86, MHC-I, and MHC-II on microglia, while there was no change in CD80 expression (Figure 3B). In addition, we isolated microglia with magnetic beads and measured the expression of microglial cytokines and chemokines by real-time qPCR. Our results showed that the expression of BDNF and IL-1β was significantly lower in the RU24969 group than in the control group. There was no significant difference in IL-6 or TNF expression between the two groups (Figure 3C). These results indicate that microglia were functionally inhibited in the OCD model.

### BDNF and Trehalose Effectively Prevented Stereotyped Behavior in the OCD Mouse Model

**Figure 4:**
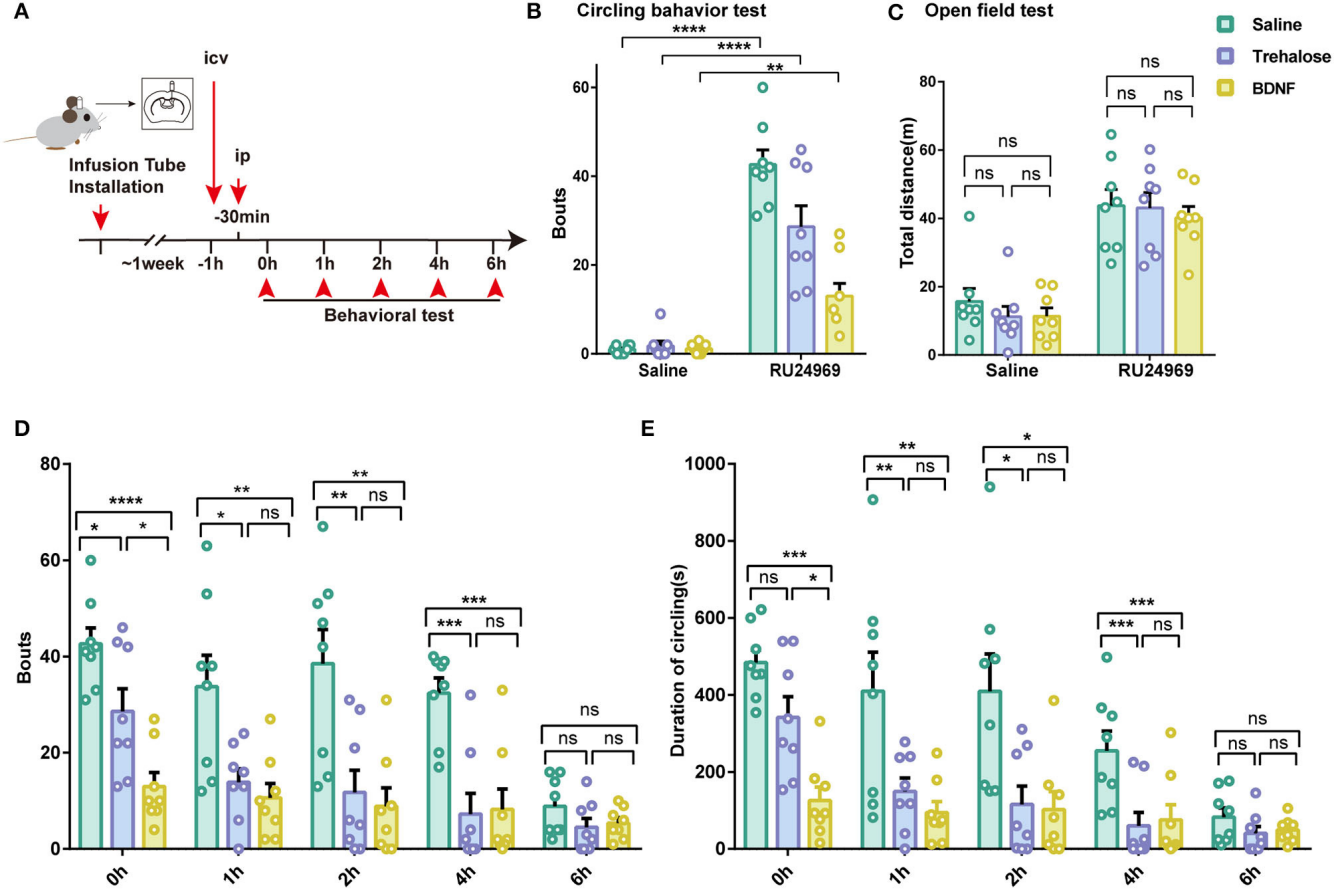
Increasing the level of BDNF in the brain effectively alleviates stereotypic behaviors. **(A)** Schematic diagram of icv injection and experimental procedure. **(B)** RU24969 could still induce obvious circling behavior after icv injection (*n* = 8 mice; ip saline+ icv saline vs. ip RU24969+icv saline: 1 ± 0.3273 vs. 42.63 ± 3.3, *t*_14_ = 12.55, *P* < 0.0001; ip saline+ icv trehalose vs. ip RU24969+icv trehalose: 1.75 ± 1.082 vs. 28.63 ± 4.698, *t*_14_ = 5.575, *P* < 0.0001; ip saline+ icv BDNF vs. ip RU24969+icv BDNF: 1.125 ± 0.3981 vs. 13 ± 2.885, *t*_14_ = 4.078, *P* < 0.01). **(C)** icv injection did not inhibit the movement of mice [*n* = 8 mice; ip saline: *F*_(2, 21)_ = 0.6426, *P* = 0.5360 > 0.05; ip RU24969: *F*_(2, 21)_ = 0.1962, *P* = 0.8233 > 0.05]. **(D,E)** Number of bouts and total duration of circling behavior were recorded at different time points after drug administration through the lateral ventricle [*n* = 8 mice; bouts: 0 h: *F*_(2, 21)_ = 15.96, *P* < 0.0001; 1 h: *F*_(2, 21)_ = 6.184, *P* < 0.01; 2 h: *F*_(2, 21)_ = 9.287, *P* < 0.01; 4 h: *F*_(2, 21)_ = 13.09, *P* < 0.001; 6 h: *F*_(2, 21)_ = 1.749, *P* = 0.1983 > 0.05. Duration: 0 h: *F*_(2, 21)_ = 13.52, *P* < 0.001; 1 h: *F*_(2, 21)_ = 9.711, *P* < 0.01; 2 h: *F*_(2, 21)_ = 6.002, *P* < 0.01; 4 h: *F*_(2, 21)_ = 13.25, *P* < 0.001; 6 h: *F*_(2, 21)_ = 1.527, *P* = 0.2402 > 0.05]. Data are presented as the mean ± SEM. Data were analyzed by two-tailed Student's *t*-test or one-way ANOVA followed by multiple comparisons with a Bonferroni correction; *P < 0.05, **P < 0.01, ***P < 0.001, ****P < 0.0001, ns stands for “not significant”.

Given that the expression of microglial BDNF was decreased and that BDNF is a crucial factor for proper cerebral function, we tested whether supplementation with BDNF or induction of BDNF expression in the brain could reduce the stereotyped behavior of mice. Previous studies have shown that trehalose can promote the synthesis and release of BDNF in the brain (Perucho et al., 2016). Therefore, we microinjected BDNF or trehalose into the lateral ventricle in control and RU24969-treated mice. We examined the behavior of the mice at different time points after intracerebral injection (Figure 4A). Our results showed that BDNF treatment significantly reduced circling behavior in RU24969-treated mice, although the total distance traveled in the open field test was not affected. Similar results were also obtained in the trehalose group (Figures 4B,C).

We further examined the time course of these effects. We started the circling behavior test 30 min after the ip injection of saline or RU24969. We found that intracerebroventricular (icv) injection of trehalose or BDNF could effectively reduce the number of bouts of circling behaviors. The effects lasted until 6 h after RU24969 treatment (Figures 4D,E). These results indicate that the administration of BDNF or the induction of BDNF expression by trehalose could effectively reduce OCD-like behaviors for a sustained period.

### Intracerebroventricular Injection of BDNF Reversed Microglial Dysfunction

**Figure 5:**
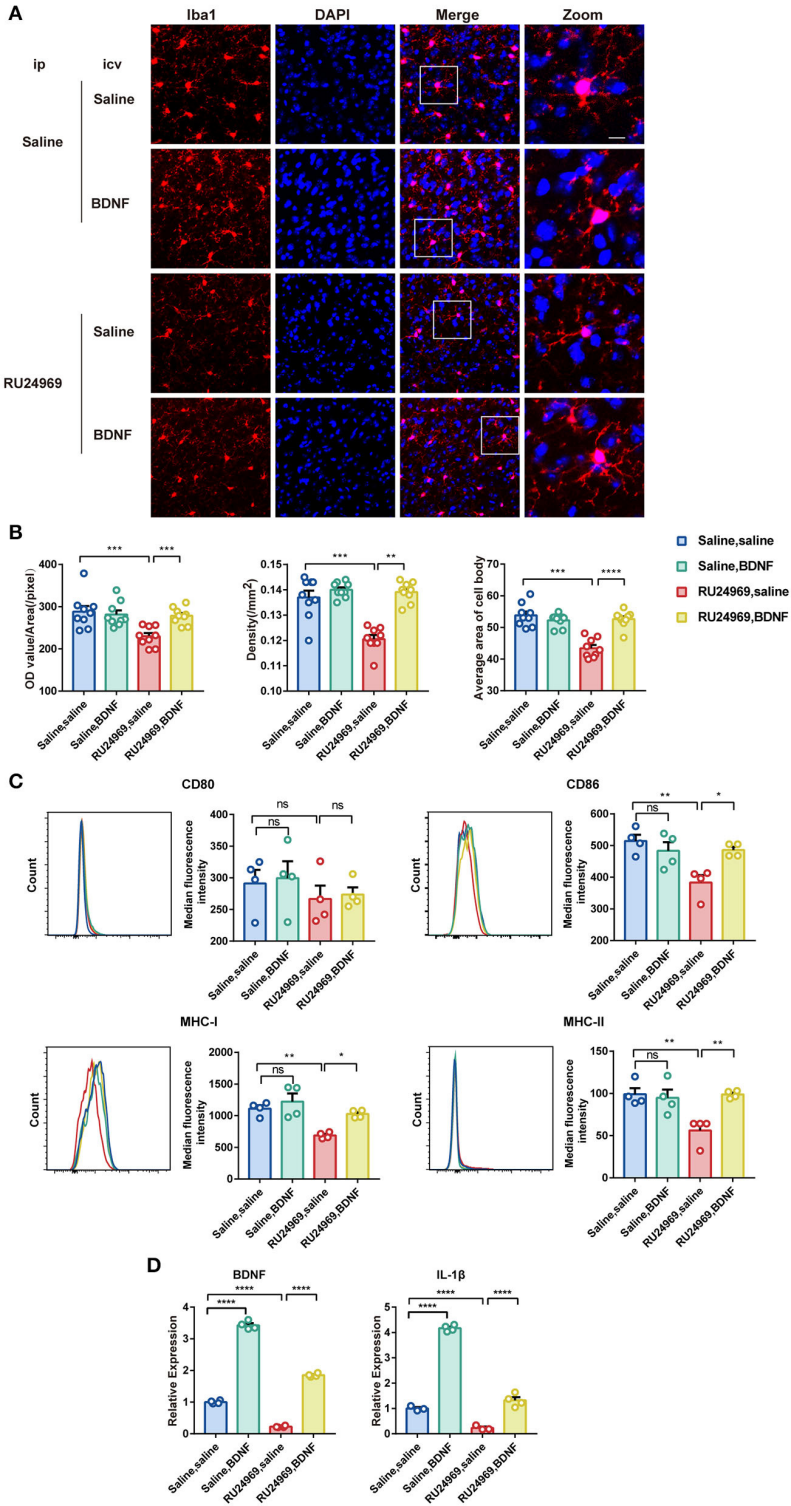
Exogenous BDNF supplementation enhanced the activity and function of microglia. **(A)** Representative image of Iba1+ microglia in the PrL after drug supplementation in the lateral ventricle. Scale bar: 10 μm. **(B)** Quantification of Iba1+ cell fluorescence, density, and cell body area in the PrL after drug supplementation in the lateral ventricle (*n* = 3 mice, 3 brain slices for each mouse. OD value: 0.1206 ± 0.001591 vs. 0.1391 ± 0.001328, *P* < 0.001; density: 229.7 ± 7.702 vs. 278.3 ± 6.629, *P* < 0.01; cell body area: 43.44 ± 0.9999 vs. 52.62 ± 0.8626, *P* < 0.0001). **(C)** Quantification of the median fluorescence intensity of CD80, CD86, MHC-I, and MHC-II in microglia in the PrL (*n* = 4 mice. CD86: 383.3 ± 23.38 vs. 486 ± 10.39, *P* < 0.05; MHC-I: 688.5 ± 24.06 vs. 1027 ± 30.43, *P* < 0.05; MHC-II: 56.18 ± 8.025 vs. 98.8 ± 2.211, *P* < 0.01). **(D)** Expression levels of BDNF and IL-1β in each group (*n* = 3 or 4 mice. Saline group: BDNF: 0.2227 ± 0.01294 vs. 1.85 ± 0.02648, *P* < 0.0001; IL-1β: 0.2302 ± 0.053 vs. 1.319 ± 0.1255, *P* < 0.0001. RU24969 group: BDNF: 1 ± 0.02475 vs. 3.427 ± 0.06455, *P* < 0.0001; IL-1β: 1 ± 0.05296 vs. 4.167 ± 0.06197, *P* < 0.0001). Data were analyzed by two-tailed Student's *t*-test or two-way ANOVA followed by multiple comparisons with a Bonferroni correction; *P < 0.05, **P < 0.01, ***P < 0.001, ****P < 0.0001, ns stands for “not significant”.

We further explored the changes in microglial activity after BDNF supplementation. Our results showed that BDNF supplementation significantly increased the OD, cell density, and average cell body area of microglia in RU24969-treated mice but did not alter those variables in the saline-treated mice (Figures 5A,B). In the flow cytometry experiments, we obtained similar results. BDNF effectively restored the expression of CD86, MHC-I, and MHC-II on the surface of microglia in the RU24969 group but did not change those variables in the control group (Figure 5C). Similarly, in the real-time qPCR experiment, BDNF promoted the expression of BDNF and IL-1β in the microglia of both RU24969 mice and control mice (Figure 5D).

These results indicate that icv injection of BDNF could effectively reverse the microglial dysfunction induced by RU24969.

## Discussion

In this study, we found that RU24969 treatment led to OCD-like behaviors in mice, accompanied by reduced microglial density and mobility, as well as reduced expression of cytokines and chemokines by microglia. The administration of BDNF or the induction of BDNF expression by trehalose completely reversed this microglial dysfunction and ameliorated OCD-like behaviors. These results indicate that microglia could be a potential therapeutic target for OCD and that BDNF could be an effective means of OCD treatment.

Microglia are the primary immune cells in the central nervous system. They are distributed throughout the parenchyma, accounting for ~10–20% of glial cells in the brain (Lynch, 2009). In the healthy mature brain under normal physiological conditions, microglia show extensive, fine processes and low cell body mobility; these cells perform surveillance, constantly scanning the surrounding environment (Nimmerjahn et al., 2005).

In this study, we demonstrated that microglia play an essential role in the acute compulsive-like behavior induced by RU24969 (O'Neill and Parameswaran, 1997; Chen et al., 2021). Previous studies on the mechanisms of RU24969-induced OCD-like behaviors mainly focused on the effects of this agent on neurons (Saudou et al., 1994). Few studies have examined the effect of RU24969 on microglia. As a 5-HT 1A/1B receptor agonist, RU24969 could also act directly on microglia because it has been shown that microglia express the 5-HT 1A receptor (Krabbe et al., 2012). In prior studies on the pathogenesis of OCD, mice with a Hoxb8 mutation showed compulsive grooming behavior similar to that of humans with OCD, linking the abnormality of microglia with the occurrence of obsessive-compulsive symptoms (Greer and Capecchi, 2002). Furthermore, almost all previous research studies have been focused on the neuroinflammatory effects of microglial activation (Attwells et al., 2017). In this study, however, we found that the morphology, dynamics, and function of microglia after RU24969 treatment indicated a *silent* state, that is, less activity than microglia display in the normal physiological state.

Previously, there was little attention to the effect of the inhibiting microglial function on neural networks and disease occurrence. In this study, we discovered that the inhibition of microglial functions might trigger compulsions. In functional imaging studies, patients with OCD had different degrees of abnormal enhancement in neuronal activity of the cortex and striatum compared with normal control. This abnormal enhancement of neuron activity is bound to the breakdown of the balance of excitation/inhibition in the central nervous system, leading to the onset of OCD (Breiter et al., 1996; Saxena and Rauch, 2000; Goodman et al., 2021). Microglia have been demonstrated to inhibit the activation of neurons (Badimon et al., 2020). Therefore, we speculated that ip injection of RU24969 inhibited the activity of microglia, significantly reduced their monitoring ability, and then reduced their inhibitory effect on the overexcitation of neurons, ultimately leading to the occurrence of rigid behaviors.

We found that RU24969 decreased the expression of BDNF after treatment. Although BDNF in the brain is mainly released by neurons, microglia are also an essential source of BDNF. Decreased release of BDNF by microglia may result in decreased overall BDNF levels, which is consistent with studies showing that the serum concentration of BDNF in patients with OCD is significantly lower than that in normal controls (Wang et al., 2011). In this study, we innovatively used BDNF supplementation to treat stereotyped behaviors in mice. In addition, we indirectly increased BDNF levels by injecting trehalose into the lateral ventricles of mice because studies have shown that trehalose can promote the synthesis and release of BDNF in the brain (Perucho et al., 2016). Both direct and indirect supplementation with BDNF effectively reduced the compulsive-like behavior induced by RU24969. Although trehalose supplementation had a lesser therapeutic effect than BDNF at the initial stage of the experiment, it still significantly ameliorated the stereotyped behaviors. These late-onset effects might be due to the delayed induction of BDNF by trehalose as the brain needs a certain amount of time to complete the synthesis and release of BDNF.

In conclusion, our study identified that a reduction in microglial activity was critical in the pathology of the RU24969-induced OCD mouse model. This result provides a new angle to investigate the pathogenesis of OCD. Furthermore, counterbalancing the previous view that activated microglia could lead to OCD (Attwells et al., 2017), our results suggested that inhibited microglia could also lead to OCD-like behaviors. Therefore, although the pathogenesis of OCD requires further study, the role of microglia should not be ignored.

There are some limitations of our study. First, as OCD is a chronic mental illness, one of its diagnostic criteria in the 10th revision of the International Classification of Diseases (ICD-10) is that obsessive-compulsive symptoms must last for at least 2 weeks (Veale and Roberts, 2014). However, the RU24969 mouse model is an acute OCD model and thus cannot simulate the actual chronic condition of OCD in humans. In addition, our study was limited to microglia and did not explore their interaction with neurons. Finally, similar to most animal studies, our study was performed in mice; there are many distinctions in neural functions between humans and mice, calling attention to the need for clinical translation research.

## Data Availability Statement

The original contributions presented in the study are included in the article/supplementary material, further inquiries can be directed to the corresponding authors.

## Ethics Statement

The animal study was reviewed and approved by Animal Care and Use Committee of Sun Yat-sen University.

## Author Contributions

SW, BL, and KS conceived the project and design of the experiments. YL and XC performed the experiments. YL, XC, CW, HZ, and LH contributed to the analysis and interpretation of the results. YL, XC, LZ, KS, and BL wrote the manuscript. All authors contributed to the article and approved the submitted version.

## Funding

This research was supported by the National Key R&D Program of China (Grants 2018YFA0108300 to BL, 2021YFF1200700 to LH), National Natural Science Foundation of China (Grants 81622016 and 31571034 to BL, 81871048 and 82071241 to LH), Guangdong Natural Science Foundation (Grants for Distinguished Young Scholar 2015A030306019 to BL, 2018B030311034 to LH), Guangdong Provincial Key R&D Programs (Grant Key Technologies for Treatment of Brain Disorders 2018B030332001 and Development of New Tools for Diagnosis and Treatment of Autism 2018B030335001 to BL and LH), and Natural Science Foundation of Guangdong (Grant 2019A1515011308 to SW).

## Conflict of Interest

The authors declare that the research was conducted in the absence of any commercial or financial relationships that could be construed as a potential conflict of interest.

## Publisher's Note

All claims expressed in this article are solely those of the authors and do not necessarily represent those of their affiliated organizations, or those of the publisher, the editors and the reviewers. Any product that may be evaluated in this article, or claim that may be made by its manufacturer, is not guaranteed or endorsed by the publisher.
